# Timed Automata Relaxation for Reachability

**DOI:** 10.1007/978-3-030-72016-2_16

**Published:** 2021-03-01

**Authors:** Jaroslav Bendík, Ahmet Sencan, Ebru Aydin Gol, Ivana Černá

**Affiliations:** 8 Eindhoven University of Technology, Eindhoven, The Netherlands; 9 Aalborg University, Aalborg East, Denmark; 10 Faculty of Informatics, Masaryk University, Brno, Czech Republic; 11 Department of Computer Engineering, Middle East Technical University, Ankara, Turkey

**Keywords:** Timed Automata, Relaxation, Design, Reachability.

## Abstract

Timed automata (TA) have shown to be a suitable formalism for modeling real-time systems. Moreover, modern model-checking tools allow a designer to check whether a TA complies with the system specification. However, the exact timing constraints of the system are often uncertain during the design phase. Consequently, the designer is able to build a TA with a correct structure, however, the timing constraints need to be tuned to make the TA comply with the specification.

In this work, we assume that we are given a TA together with an existential property, such as reachability, that is not satisfied by the TA. We propose a novel concept of a minimal sufficient reduction (MSR) that allows us to identify the minimal set *S* of timing constraints of the TA that needs to be tuned to meet the specification. Moreover, we employ mixed-integer linear programming to actually find a tuning of *S* that leads to meeting the specification.

## 1 Introduction

A timed automaton (TA) [4] is a finite automaton extended with a set of real-time variables, called clocks, which capture the time. The clocks enrich the semantics and the constraints on the clocks restrict the behavior of the automaton, which are particularly important in modeling time-critical systems. The examples of TA models of critical systems include scheduling of real-time systems [30,29,33], medical devices [43,38], and rail-road crossing systems [52].

Model-checking methods allow for verifying whether a given TA meets a given system specification. Contemporary model-checking tools, such as UPPAAL [17] or Imitator [9], have proved to be practically applicable on various industrial case studies [17,10,34]. Unfortunately, during the system design phase, the system information is often incomplete. A designer is often able to build a TA with correct structure, i.e., exactly capturing locations and transitions of the modeled system, however the exact clock (timing) constraints that enable/trigger the transitions are uncertain. Thus, the produced TA often does not meet the specification (i.e., it does not pass the model-checking) and it needs to be fixed. If the specification declares universal properties, e.g., safety or unavoidability, that need to hold on each trace of the TA, a model-checker either returns "yes", or it returns "no" and generates a trace along which the property is violated. This trace can be used to repair the model in an automated way [42]. However, in the case of existential properties, such as reachability, the property has to hold on a trace of the TA. The model-checker either returns "yes" and generates a witness trace satisfying the property, or returns just "no" and does not provide any additional information that would help the designer to correct the TA.

**Contribution.** In this paper, we study the following problem: given a timed automaton $\mathcal{A}$ and a reachability property that is not satisfied by $\mathcal{A}$, relax clock constraints of $\mathcal{A}$ such that the resultant automaton $\mathcal{A}'$ satisfies the reachability property. Moreover, the goal is to minimize the number of the relaxed clock constraints and, secondary, also to minimize the overall change of the timing constants used in the clock constraints. We propose a two step solution for this problem. In the first step, we identify a *minimal sufficient reduction (MSR)* of $\mathcal{A}$, i.e., an automaton $\mathcal{A}''$ that satisfies the reachability property and originates from $\mathcal{A}$ by removing only a minimal necessary set of clock constraints. In the second step, instead of completely removing the clock constraints, we employ mixed integer linear programming (MILP) to find a minimal relaxation of the constraints that leads to a satisfaction of the reachability property along a witness path.

The underlying assumption is that during the design the most suitable timing constants reflecting the system properties are defined. Thus, our goal is to generate a TA satisfying the reachability property by changing a minimum number of timing constants. Some of the constraints of the initial TA can be strict (no relaxation is possible), which can easily be integrated to the proposed solution. Thus, the proposed method can be viewed as a way to handle design uncertainties: develop a TA $\mathcal{A}$ in a best-effort basis and apply our algorithm to find a $\mathcal{A}'$ that is *as close as* possible to $\mathcal{A}$ and satisfies the given reachability property.

**Related Work.** Another way to handle uncertainties about timing constants is to build a *parametric* timed automaton (PTA), i.e., a TA where clock constants can be represented with parameters. Subsequently, a parameter synthesis tool, such as [46,9,26], can be used to find suitable values of the parameters for which the resultant TA satisfies the specification. However, most of the parameter synthesis problems are undecidable [6]. While symbolic algorithms without termination guarantees exist for some subclasses [25,39,12], these algorithms are computationally very expensive compared to model checking (see [5]). Moreover, minimizing the number of modified clock constraints is not straightforward.

A related TA repair problem has been studied in a recent work [7], where the authors also assumed that some of the constraints are incorrect. To repair the TA, they parametrized the initial TA and generated parameters by analyzing traces of the TA. However, the authors [7] do not focus on repairing the TA w.r.t. reachability properties as we do. Instead, their goal is to make the TA compliant with an oracle that decides if a trace of the TA belongs to a system or not. Thus, their approach cannot handle reachability properties. Furthermore in [7], the total change of the timing constraints is minimized, while we primarily minimize the number of changed constraints, then the total change.

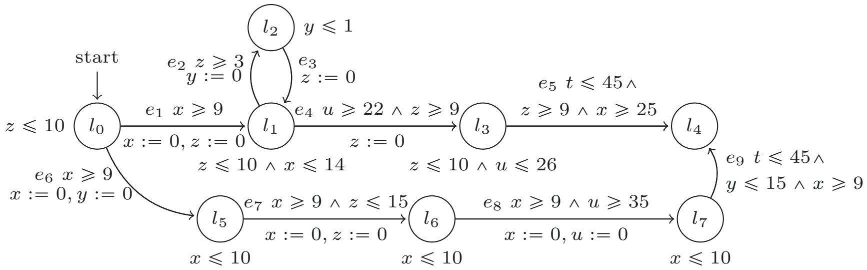

**Fig. 1.** An example of a timed automaton.

## 2 Preliminaries and Problem Formulation

### 2.1 Timed Automata

A *timed automaton* (TA) [3,4,44] is a finite-state machine extended with a finite set $C$ of real-valued clocks. A *clock* $x \in C$ measures the time spent after its last reset. In a TA, clock constraints are defined for locations (states) and transitions. A *simple clock constraint* is defined as $x - y \sim c$ where $x, y \in C \cup \{0\}$, $\sim \in \{<, \leqslant\}$ and $c \in \mathbb{Z} \cup \{\infty\}$.[3] Simple clock constraints and constraints obtained by combining these with conjunction operator ($\wedge$) are called *clock constraints*. The sets of simple and all clock constraints are denoted by $\Phi_S(C)$ and $\Phi(C)$, respectively. For a clock constraint $\phi \in \Phi(C)$, $\mathcal{S}(\phi)$ denotes the simple constraints from $\phi$, e.g., $\mathcal{S}(x - y < 10 \wedge y \leqslant 20) = \{x - y < 10, y \leqslant 20\}$. A *clock valuation* $v : C \to \mathbb{R}_+$ assigns non-negative real values to each clock. The notation $v \models \phi$ denotes that the clock constraint $\phi$ evaluates to true when each clock $x$ is replaced with $v(x)$. For a clock valuation $v$ and $d \in \mathbb{R}_+$, $v + d$ is the clock valuation obtained by *delaying* each clock by $d$, i.e., $(v + d)(x) = v(x) + d$ for each $x \in C$. For $\lambda \subseteq C$, $v[\lambda := 0]$ is the clock valuation obtained after *resetting* each clock from $\lambda$, i.e., $v[\lambda := 0](x) = 0$ for each $x \in \lambda$ and $v[\lambda := 0](x) = v(x)$ for each $x \in C \backslash \lambda$.

**Definition 1 (Timed Automata).** *A timed automaton $\mathcal{A} = (L, l_0, C, \Delta, Inv)$ is a tuple, where $L$ is a finite set of locations, $l_0 \in L$ is the initial location, $C$ is a finite set of clocks, $\Delta \subseteq L \times 2^C \times \Phi(C) \times L$ is a finite transition relation, and $Inv : L \to \Phi(C)$ is an invariant function.*

For a transition $e = (l_s, \lambda, \phi, l_t) \in \Delta$, $l_s$ is the source location, $l_t$ is the target location, $\lambda$ is the set of clocks reset on $e$ and $\phi$ is the guard (i.e., a clock constraint) tested for enabling $e$. The semantics of a TA is given by a labelled transition system (LTS). An LTS is a tuple $\mathcal{T} = (S, s_0, \Sigma, \to)$, where $S$ is a set of states, $s_0 \in S$ is an initial state, $\Sigma$ is a set of symbols, and $\to \subseteq S \times \Sigma \times S$ is a transition relation. A transition $(s, a, s') \in \to$ is also shown as $s \xrightarrow{a} s'$.

**Definition 2 (LTS semantics for TA).** *Given a TA $\mathcal{A} = (L, l_0, C, \Delta, Inv)$, the labelled transition system $T(\mathcal{A}) = (S, s_0, \Sigma, \to)$ is defined as follows:*

[3] Simple constraints are only defined as upper bounds to ease the presentation. This definition is not restrictive since $x - y \geqslant c$ and $x \geqslant c$ are equivalent to $y - x \leqslant -c$ and $0 - x \leqslant -c$, respectively. A similar argument holds for strict inequality ($>$).

– $S = \{(l, v) \mid l \in L, v \in \mathbb{R}_+^{|C|}, v \models Inv(l)\}$,
– $s_0 = (l_0, \boldsymbol{0})$, *where* $\boldsymbol{0}(x) = 0$ *for each* $x \in C$,
– $\Sigma = \{act\} \cup \mathbb{R}_+$, *and*
– *the transition relation* $\rightarrow$ *is defined by the following rules:*
  - *delay transition:* $(l, v) \xrightarrow{d} (l, v + d)$ *if* $v + d \models Inv(l)$
  - *discrete transition:* $(l, v) \xrightarrow{act} (l', v')$ *if there exists* $(l, \lambda, \phi, l') \in \Delta$ *such that* $v \models \phi$, $v' = v[\lambda := 0]$, *and* $v' \models Inv(l')$.

The notation $s \rightarrow_d s'$ is used to denote a delay transition of duration $d$ followed by a discrete transition from $s$ to $s'$, i.e., $s \xrightarrow{d} s \xrightarrow{act} s'$. A run $\rho$ of $\mathcal{A}$ is either a finite or an infinite alternating sequence of delay and discrete transitions, i.e., $\rho = s_0 \rightarrow_{d_0} s_1 \rightarrow_{d_1} s_2 \rightarrow_{d_2} \cdots$. The set of all runs of $\mathcal{A}$ is denoted by $[[\mathcal{A}]]$.

A path $\pi$ of $\mathcal{A}$ is an interleaving sequence of locations and transitions, $\pi = l_0, e_1, l_1, e_2, \ldots$, where $e_{i+1} = (l_i, \lambda_{i+1}, \phi_{i+1}, l_{i+1}) \in \Delta$ for each $i \geqslant 0$. A path $\pi = l_0, e_1, l_1, e_2, \ldots$ is said to be *realizable* if there exists a delay sequence $d_0, d_1, \ldots$ such that $(l_0, \mathbf{0}) \rightarrow_{d_0} (l_1, v_1) \rightarrow_{d_1} (l_1, v_2) \rightarrow_{d_2} \cdots$ is a run of $\mathcal{A}$ and for every $i \geqslant 1$, the $i$th discrete transition is taken according to $e_i$, i.e., $e_i = (l_{i-1}, \lambda_i, \phi_i, l_i)$, $v_{i-1} + d_{i-1} \models \phi_i$, $v_i = (v_{i-1} + d_{i-1})[\lambda_i := 0]$ and $v_i \models Inv'(l_i)$.

Given a TA $\mathcal{A}$, a subset $L_T \subset L$ of its locations is *reachable* on $\mathcal{A}$ if there exists $\rho = (l_0, \mathbf{0}) \rightarrow_{d_0} (l_1, v_1) \rightarrow_{d_1} \ldots \rightarrow_{d_{n-1}} (l_n, v_n) \in [[\mathcal{A}]]$ such that $l_n \in L_T$; otherwise, $L_T$ is *unreachable*. The reachability problem is decidable and implemented in various verification tools, e.g., [17,9]. The verifier either returns "No" when the location is unreachable, or it generates a run (witness) reaching the set $L_T$.

*Example 1.* Figure 1 illustrates a TA with 8 locations: $\{l_0, \ldots, l_7\}$, 9 transitions: $\{e_1, \ldots, e_9\}$, an initial location $l_0$, and an unreachable set of locations $L_T = \{l_4\}$.

### 2.2 Timed Automata Relaxations and Reductions

For a timed automaton $\mathcal{A} = (L, l_0, C, \Delta, Inv)$, the set of pairs of transition and associated simple constraints is defined in (1) and the set of pairs of location and associated simple constraints is defined in (2).

$$\Psi(\Delta) = \{(e, \varphi) \mid e = (l_s, \lambda, \phi, l_t) \in \Delta, \varphi \in \mathcal{S}(\phi)\} \tag{1}$$

$$\Psi(Inv) = \{(l, \varphi) \mid l \in L, \varphi \in \mathcal{S}(Inv(l))\} \tag{2}$$

**Definition 3 (constraint-relaxation).** *Let* $\phi \in \Phi(C)$ *be a constraint over* $C$, $\Theta \subseteq \mathcal{S}(\phi)$ *be a subset of its simple constraints and* $\mathbf{r} : \Theta \rightarrow \mathbb{N} \cup \{\infty\}$ *be a positive valued relaxation valuation. The relaxed constraint is defined as:*

$$R(\phi, \Theta, \mathbf{r}) = \left( \bigwedge_{\varphi \in \mathcal{S}(\phi) \setminus \Theta} \varphi \right) \wedge \left( \bigwedge_{\varphi = x - y \sim c \in \Theta} x - y \sim c + \mathbf{r}(\varphi) \right) \tag{3}$$

Intuitively, $R(\phi, \Theta, \mathbf{r})$ relaxes only the thresholds of simple constraints from $\Theta$ with respect to $\mathbf{r}$, e.g., $R(x - y \leqslant 10 \wedge y < 20, \{y < 20\}, \mathbf{r}) = x - y \leqslant 10 \wedge y < 23$, where $\mathbf{r}(y < 20) = 3$. Setting a threshold to $\infty$ implies removing the corresponding simple constraint, e.g., $R(x - y \leqslant 10 \wedge y < 20, \{y < 20\}, \mathbf{r}) = x - y \leqslant 10$, where $\mathbf{r}(y < 20) = \infty$. Note that $R(\phi, \Theta, \mathbf{r}) = \phi$ when $\Theta$ is empty.

**Definition 4** (**$(D, I, \mathbf{r})$-relaxation).** *Let $\mathcal{A} = (L, l_0, C, \Delta, Inv)$ be a TA, $D \subseteq \Psi(\Delta)$ and $I \subseteq \Psi(Inv)$ be transition and location constraint sets, and $\mathbf{r} : D \cup I \to \mathbb{N} \cup \{\infty\}$ be a positive valued relaxation valuation. The $(D, I, \mathbf{r})$-relaxation of $\mathcal{A}$, denoted $\mathcal{A}_{<D,I,\mathbf{r}>}$, is a TA $\mathcal{A}' = (L', l'_0, C', \Delta', Inv')$ such that:*

- *$L = L'$, $l_0 = l'_0$, $C = C'$, and*
- *$\Delta'$ originates from $\Delta$ by relaxing $D$ via $\mathbf{r}$. For $e = (l_s, \lambda, \phi, l_t) \in \Delta$, let $D|_e = \{\varphi \mid (e, \varphi) \in D\}$, and let $\mathbf{r}|_e(\varphi) = \mathbf{r}(e, \varphi)$, then $\Delta' = \{(l_s, \lambda, R(\phi, D|_e, \mathbf{r}|_e), l_t) \mid e = (l_s, \lambda, \phi, l_t) \in \Delta\}$*
- *$Inv'$ originates from $Inv$ by relaxing $I$ via $\mathbf{r}$. For $l \in L$, let $I|_l = \{\varphi \mid (l, \varphi) \in I\}$, and $\mathbf{r}|_l(\varphi) = \mathbf{r}(l, \varphi)$, then $Inv'(l) = R(Inv(l), I|_l, \mathbf{r}|_l)$.*

Intuitively, the TA $\mathcal{A}_{<D,I,\mathbf{r}>}$ emerges from $\mathcal{A}$ by relaxing the guards of the transitions from the set $D$ and relaxing invariants of the locations from $I$ with respect to $\mathbf{r}$. In the special case of setting the threshold of each constraint from $D$ and $I$ to $\infty$, i.e., when $\mathbf{r}(a) = \infty$ for each $a \in D \cup I$, the corresponding simple constraints are effectively removed, which is called a $(D,I)$-reduction and denoted by $\mathcal{A}_{<D,I>}$. Note that $\mathcal{A} = \mathcal{A}_{<\emptyset,\emptyset>}$.

**Proposition 1.** *Let $\mathcal{A} = (L, l_0, C, \Delta, Inv)$ be a timed automaton, $D \subseteq \Psi(\Delta)$ and $I \subseteq \Psi(Inv)$ be sets of simple guard and invariant constraints, and $\mathbf{r} : D \cup I \to \mathbb{N} \cup \{\infty\}$ be a relaxation valuation. Then $[[\mathcal{A}]] \subseteq [[\mathcal{A}_{<D,I,\mathbf{r}>}]]$.*

*Proof.* Observe that for a clock constraint $\phi \in \Phi(C)$, a subset of its simple constraints $\Theta \subseteq \mathcal{S}(\phi)$, a relaxation valuation $\mathbf{r}'$ for $\Theta$, and the relaxed constraint $R(\phi, \Theta, \mathbf{r}')$ as in Definition 3, it holds that for any clock valuation $v : v \models \phi \implies v \models R(\phi, \Theta, \mathbf{r}')$. Now, consider a run $\rho = (l_0, \mathbf{0}) \to_{d_0} (l_1, v_1) \to_{d_1} (l_2, v_2) \to_{d_2} \cdots \in [[\mathcal{A}]]$. Let $\pi = l_0, e_1, l_1, e_2, \ldots$ with $e_i = (l_{i-1}, \lambda_i, \phi_i, l_i) \in \Delta$ for each $i \geqslant 1$ be the path realized as $\rho$ via delay sequence $d_0, d_1, \ldots$. By Definition 4 for each $(l, \lambda, \phi, l') \in \Delta$, there is $(l, \lambda, R(\phi, D|_e, \mathbf{r}|_e), l') \in \Delta'$. We define a path induced by $\pi$ on $\mathcal{A}_{<D,I,\mathbf{r}>}$ as:

$$M(\pi) = l_0, (l_0, \lambda_1, R(\phi_1, D|_{e_1}, \mathbf{r}|_{e_1}), l_1), l_1, (l_1, \lambda_2, R(\phi_2, D|_{e_2}, \mathbf{r}|_{e_2}), l_2), \ldots \quad (4)$$

For each $i = 0, \ldots, n-1$ it holds that $v_i \models R(Inv(l_i), D|_{l_i}, \mathbf{r}|_{l_i})$, $v_i + d_i \models R(Inv(l_i), D|_{l_i}, \mathbf{r}|_{l_i})$ and $v_i + d_i \models R(\phi_{i+1}, D|_{e_{i+1}}, \mathbf{r}|_{e_{i+1}})$. Thus $M(\pi)$ is realizable on $\mathcal{A}_{<D,I,\mathbf{r}>}$ via the same delay sequence and $\rho \in [[\mathcal{A}_{<D,I,\mathbf{r}>}]]$. As $\rho \in [[\mathcal{A}]]$ is arbitrary, we conclude that $[[\mathcal{A}]] \subseteq [[\mathcal{A}_{<D,I,\mathbf{r}>}]]$.

### 2.3 Problem Statement

*Problem 1.* Given a TA $\mathcal{A} = (L, l_0, C, \Delta, Inv)$ and a set of target locations $L_T \subset L$ that is unreachable on $\mathcal{A}$, find a $(D, I, \mathbf{r})$-relaxation $\mathcal{A}_{<D,I,\mathbf{r}>}$ of $\mathcal{A}$ such that $L_T$ is reachable on $\mathcal{A}_{<D,I,\mathbf{r}>}$. Moreover, the goal is to identify a $(D, I, \mathbf{r})$-relaxation that minimizes the number $|D \cup I|$ of relaxed constraints, and, secondary, we tend to minimize the overall change of the clock constraints $\sum_{c \in D \cup I} \mathbf{r}(c)$.

We propose a two step solution to this problem. In the first step, we identify a subset $D \cup I$ of the simple constraints $\Psi(\Delta) \cup \Psi(Inv)$ such that $L_T$ is reachable on the $(D, I)$-reduction $\mathcal{A}_{<D,I>}$ and $|D \cup I|$ is minimized. Consequently, we can obtain a witness path of the reachability on $\mathcal{A}_{<D,I>}$ from the verifier. The path would be realizable on $\mathcal{A}$ if we remove the constraints $D \cup I$. In the second step, instead of completely removing the constraints $D \cup I$, we find a relaxation valuation $\mathbf{r} : D \cup I \to \mathbb{N} \cup \{\infty\}$ such that the path found in the first step is realizable on $\mathcal{A}_{<D,I,\mathbf{r}>}$. To find $\mathbf{r}$, we introduce relaxation parameters for constraints in $D \cup I$. Subsequently, we solve an MILP problem to find a valuation of the parameters, i.e., $\mathbf{r}$, that makes the path realizable on $\mathcal{A}_{<D,I,\mathbf{r}>}$ and minimizes $\sum_{c \in D \cup I} \mathbf{r}(c)$. Note that it might be the case that the reduction $\mathcal{A}_{<D,I>}$ contains multiple realizable paths that lead to $L_T$, and another path might result in a smaller overall change. Also, there might exist another candidate subset $D' \cup I'$ with $|D' \cup I'| = |D \cup I|$ that would lead to a smaller overall change. While our approach can be applied to a number of paths and a number of candidate subsets $D \cup I$, processing all of them can be practically intractable.

## 3 Minimal Sufficient $(D,I)$-Reductions

Throughout this section, we simply write a *reduction* when talking about a $(D,I)$-reduction of $\mathcal{A}$. To name a reduction, we either simply use capital letters, e.g., $M, N, K$, or we use the notation $\mathcal{A}_{<D,I>}$ to also specify the sets $D, I$ of simple clock constraints. Given a reduction $N = \mathcal{A}_{<D,I>}$, $|N|$ denotes the cardinality $|D \cup I|$. Furthermore, $\mathcal{R}_{\mathcal{A}}$ denotes the set of all reductions. We define a partial order relation $\sqsubseteq$ on $\mathcal{R}_{\mathcal{A}}$ as $\mathcal{A}_{<D,I>} \sqsubseteq \mathcal{A}_{<D',I'>}$ iff $D \cup I \subseteq D' \cup I'$. Similarly, we write $\mathcal{A}_{<D,I>} \sqsubsetneq \mathcal{A}_{<D',I'>}$ iff $D \cup I \subsetneq D' \cup I'$. We say that a reduction $\mathcal{A}_{<D,I>}$ is a *sufficient reduction* (w.r.t. $\mathcal{A}$ and $L_T$) iff $L_T$ is reachable on $\mathcal{A}_{<D,I>}$; otherwise, $\mathcal{A}_{<D,I>}$ is an *insufficient reduction*. Crucial observation for our work is that the property of being a sufficient reduction is monotone w.r.t. the partial order:

**Proposition 2.** *Let $\mathcal{A}_{<D,I>}$ and $\mathcal{A}_{<D',I'>}$ be reductions such that $\mathcal{A}_{<D,I>} \sqsubseteq \mathcal{A}_{<D',I'>}$. If $\mathcal{A}_{<D,I>}$ is sufficient then $\mathcal{A}_{<D',I'>}$ is also sufficient.*

*Proof.* Note that $\mathcal{A}_{<D',I'>}$ is a $(D' \backslash D, I' \backslash I)$-reduction of $\mathcal{A}_{<D,I>}$. By Proposition 1, $[[\mathcal{A}_{<D,I>}]] \subseteq [[\mathcal{A}_{<D',I'>}]]$, i.e., the run of $\mathcal{A}_{<D,I>}$ that witnesses the reachability of $L_T$ is also a run of $\mathcal{A}_{<D',I'>}$.

**Definition 5 (MSR).** *A sufficient reduction $\mathcal{A}_{<D,I>}$ is a* minimal sufficient reduction (MSR) *iff there is no $c \in D \cup I$ such that the reduction $\mathcal{A}_{<D \backslash \{c\}, I \backslash \{c\}>}$ is sufficient. Equivalently, due to Proposition 2, $\mathcal{A}_{<D,I>}$ is an MSR iff there is no sufficient reduction $\mathcal{A}_{<D',I'>}$ such that $\mathcal{A}_{<D',I'>} \sqsubsetneq \mathcal{A}_{<D,I>}$.*

Recall that a reduction $\mathcal{A}_{<D,I>}$ is determined by $D \subseteq \Psi(\Delta)$ and $I \subseteq \Psi(Inv)$. Consequently, $|\mathcal{R}_{\mathcal{A}}| = 2^{|\Psi(\Delta) \cup \Psi(Inv)|}$. Moreover, there can be up to $\binom{k}{k/2}$ MSRs where $k = |\Psi(\Delta) \cup \Psi(Inv)|$ (see Sperner's theorem [51]). Also note, that the *minimality* of a reduction does not mean a *minimum* number of simple clock

**Algorithm 1:** Minimum MSR Extraction Scheme

$N \leftarrow \mathcal{A}_{<\Psi(\Delta),\Psi(Inv)>}$; $\mathcal{M} \leftarrow \emptyset$; $\mathcal{I} \leftarrow \emptyset$
**while** $N \neq$ `null` **do**
  $M,\mathcal{I} \leftarrow$ `shrink(`$N$,$\mathcal{I}$`)` // Algorithm 2
  $\mathcal{M} \leftarrow \mathcal{M} \cup \{M\}$
  $N,\mathcal{I} \leftarrow$ `findSeed(`$M$, $\mathcal{M}$, $\mathcal{I}$`)` // Algorithm 3
**return** $M$

constraints that are reduced by the reduction; there can exist two MSRs, $M$ and $N$, such that $|M| < |N|$. Since our overall goal is to relax $\mathcal{A}$ as little as possible, we identify a *minimum* MSR, i.e., an MSR $M$ such that there is no MSR $M'$ with $|M'| < |M|$, and then use the minimum MSR for the MILP part (Section 4) of our overall approach. There can be also up to $\binom{k}{k/2}$ minimum MSRs.

*Example 2.* Assume the TA $\mathcal{A}$ and $L_T = \{l_4\}$ from Example 1 (Fig. 1). There are 24 MSRs and 4 of them are minimum. For example, $\mathcal{A}_{<D,I>}$ with $D = \{(e_5, x \geqslant 25)\}$ and $I = \{(l_3, u \leqslant 26)\}$ is a minimum MSR, and $\mathcal{A}_{<D',I'>}$ with $D' = \{(e_9, y \leqslant 15), (e_7, z \leqslant 15)\}$ and $I' = \{(l_6, x \leqslant 10)\}$ is a non-minimum MSR.

### 3.1 Base Scheme For Computing a Minimum MSR

Algorithm 1 shows a high-level scheme of our approach for computing a minimum MSR. The algorithm iteratively identifies an ordered set of MSRs, $|M_1| > |M_2| > \cdots > |M_k|$, such that the last MSR $M_k$ is a minimum MSR. Each of the MSRs, say $M_i$, is identified in two steps. First, the algorithm finds a *seed*, i.e., a reduction $N_i$ such that $N_i$ is sufficient and $|N_i| < |M_{i-1}|$. Second, the algorithm *shrinks* $N_i$ into an MSR $M_i$ such that $M_i \sqsubseteq N_i$ (and thus $|M_i| \leqslant |N_i|$). The initial seed $N_1$ is $\mathcal{A}_{<\Psi(\Delta),\Psi(Inv)>}$, i.e., the reduction that removes all simple clock constraints (which makes all locations of $\mathcal{A}$ trivially reachable). Once there is no sufficient reduction $N_i$ with $|N_i| < |M_{i-1}|$, we know that $M_{i-1} = M_k$ is a minimum MSR.

Note that the algorithm also maintains two auxiliary sets, $\mathcal{M}$ and $\mathcal{I}$, to store all identified MSRs and insufficient reductions, respectively. The two sets are used during the process of finding and shrinking a seed which we describe below.

### 3.2 Shrinking a Seed

Our approach for shrinking a seed $N$ into an MSR $M$ is based on two concepts: a *critical simple clock constraint* and a *reduction core.*

**Definition 6 (critical constraint).** *Given a sufficient reduction $\mathcal{A}_{<D,I>}$, a simple clock constraint $c$ is* critical *for $\mathcal{A}_{<D,I>}$ iff $\mathcal{A}_{<D\setminus\{c\},I\setminus\{c\}>}$ is insufficient.*

**Proposition 3.** *If $c \in D \cup I$ is critical for a sufficient reduction $\mathcal{A}_{<D,I>}$ then $c$ is critical for every sufficient reduction $\mathcal{A}_{<D',I'>}$, $\mathcal{A}_{<D',I'>} \sqsubseteq \mathcal{A}_{<D,I>}$. Moreover, by Definitions 5 and 6, $\mathcal{A}_{<D,I>}$ is an MSR iff every $c \in D \cup I$ is* critical *for $\mathcal{A}_{<D,I>}$.*

**Algorithm 2:** `shrink`($\mathcal{A}_{<D,I>}, \mathcal{I}$)

$X \leftarrow \emptyset$
**while** $(D \cup I) \neq X$ **do**
  $c \leftarrow$ pick a simple clock constraint from $(D \cup I) \setminus X$
  **if** $\mathcal{A}_{<D\setminus\{c\},I\setminus\{c\}>} \notin \mathcal{I}$ ***and*** $\mathcal{A}_{<D\setminus\{c\},I\setminus\{c\}>}$ *is sufficient* **then**
    $\rho \leftarrow$ a witness run of the sufficiency of $\mathcal{A}_{<D\setminus\{c\},I\setminus\{c\}>}$
    $\mathcal{A}_{<D,I>} \leftarrow$ the reduction core of $\mathcal{A}_{<D\setminus\{c\},I\setminus\{c\}>}$ w.r.t. $\rho$
  **else**
    $X \leftarrow X \cup \{c\}$
    $\mathcal{I} \leftarrow \mathcal{I} \cup \{N \in \mathcal{R}_{\mathcal{A}} \mid N \sqsubseteq \mathcal{A}_{<D\setminus\{c\},I\setminus\{c\}>}\}$
**return** $\mathcal{A}_{<D,I>}, \mathcal{I}$

*Proof.* By contradiction, assume that $c$ is critical for $\mathcal{A}_{<D,I>}$ but not for $\mathcal{A}_{<D',I'>}$, i.e., $\mathcal{A}_{<D\setminus\{c\},I\setminus\{c\}>}$ is insufficient and $\mathcal{A}_{<D'\setminus\{c\},I'\setminus\{c\}>}$ is sufficient. As $\mathcal{A}_{<D',I'>} \sqsubseteq \mathcal{A}_{<D,I>}$, we have $\mathcal{A}_{<D'\setminus\{c\},I'\setminus\{c\}>} \sqsubseteq \mathcal{A}_{<D\setminus\{c\},I\setminus\{c\}>}$. By Proposition 2, if the reduction $\mathcal{A}_{<D'\setminus\{c\},I'\setminus\{c\}>}$ is sufficient then $\mathcal{A}_{<D\setminus\{c\},I\setminus\{c\}>}$ is also sufficient.

**Definition 7 (reduction core).** *Let $\mathcal{A}_{<D,I>}$ be a sufficient reduction, $\rho$ a witness run of the sufficiency (i.e., reachability of $L_T$ on $\mathcal{A}_{<D,I>}$), and $\pi$ the path corresponding to $\rho$. Futhermore, let $M(\pi) = l_0, e_1, \ldots, e_n, l_n$ be the path corresponding to $\pi$ on the original TA $\mathcal{A}$ defined as in (4). The* reduction core *of $\mathcal{A}_{<D,I>}$ w.r.t. $\rho$ is the reduction $\mathcal{A}_{<D',I'>}$ where $D' = \{(e,\varphi) \mid (e,\varphi) \in D \wedge e = e_i$ for some $1 \leqslant i \leqslant n\}$ and $I' = \{(l,\varphi) \mid (l,\varphi) \in I \wedge l = l_i$ for some $0 \leqslant l \leqslant n\}$.*

Intuitively, the reduction core of $\mathcal{A}_{<D,I>}$ w.r.t. $\rho$ reduces from $\mathcal{A}$ only the simple clock constraints that appear on the witness path in $\mathcal{A}$.

**Proposition 4.** *Let $\mathcal{A}_{<D,I>}$ be a sufficient reduction, $\rho$ the witness of reachability of $L_T$ on $\mathcal{A}_{<D,I>}$, and $\mathcal{A}_{<D',I'>}$ the reduction core of $\mathcal{A}_{<D,I>}$ w.r.t. $\rho$. Then $\mathcal{A}_{<D',I'>}$ is a sufficient reduction and $\mathcal{A}_{<D',I'>} \sqsubseteq \mathcal{A}_{<D,I>}$.*

*Proof.* By Definition 7, $D' \subseteq D$ and $I' \subseteq I$, thus $\mathcal{A}_{<D',I'>} \sqsubseteq \mathcal{A}_{<D,I>}$. As for the sufficiency of $\mathcal{A}_{<D',I'>}$, we only sketch the proof. Intuitively, both $\mathcal{A}_{<D,I>}$ and $\mathcal{A}_{<D',I'>}$ originate from $\mathcal{A}$ by only removing some simple clock constraints ($D \cup I$, and $D' \cup I'$, respectively), i.e., the graph structure of $\mathcal{A}_{<D,I>}$ and $\mathcal{A}_{<D',I'>}$ is the same, however, some corresponding paths of $\mathcal{A}_{<D,I>}$ and $\mathcal{A}_{<D',I'>}$ differ in the constraints that appear on the paths. By Definition 7, the path $\pi$ that corresponds to the witness run $\rho$ of $\mathcal{A}_{<D,I>}$ is also a path of $\mathcal{A}_{<D',I'>}$. Since realizability of a path depends only on the constraints along the path, if $\pi$ is realizable on $\mathcal{A}_{<D,I>}$ then $\pi$ is also realizable on $\mathcal{A}_{<D',I'>}$.

Our approach for shrinking a sufficient reduction $N$ is shown in Algorithm 2. The algorithm iteratively maintains a sufficient reduction $\mathcal{A}_{<D,I>}$ and a set $X$ of known critical constraints for $\mathcal{A}_{<D,I>}$. Initially, $\mathcal{A}_{<D,I>} = N$ and $X = \emptyset$. In each iteration, the algorithm picks a simple clock constraint $c \in (D \cup$

$I)\backslash X$ and checks the reduction $\mathcal{A}_{<D\backslash\{c\},I\backslash\{c\}>}$ for sufficiency. If $\mathcal{A}_{<D\backslash\{c\},I\backslash\{c\}>}$ is insufficient, the algorithm adds $c$ to $X$. Otherwise, if $\mathcal{A}_{<D\backslash\{c\},I\backslash\{c\}>}$ is sufficient, the algorithm obtains a witness run $\rho$ of the sufficiency from the verifier and reduces $\mathcal{A}_{<D,I>}$ to the corresponding reduction core. The algorithm terminates when $(D \cup I) = X$. An invariant of the algorithm is that every $c \in X$ is critical for $\mathcal{A}_{<D,I>}$. Thus, when $(D \cup I) = X$, $\mathcal{A}_{<D,I>}$ is an MSR (Proposition 3).

Note that the algorithm also uses the set $\mathcal{I}$ of known insufficient reductions. In particular, before calling a verifier to check a reduction for sufficiency (line 4), the algorithm first checks (in a lazy manner) whether the reduction is already known to be insufficient. Also, whenever the algorithm determines a reduction $\mathcal{A}_{<D\backslash\{c\},I\backslash\{c\}>}$ to be insufficient, it adds $\mathcal{A}_{<D\backslash\{c\},I\backslash\{c\}>}$ and every $N$, $N \sqsubseteq \mathcal{A}_{<D\backslash\{c\},I\backslash\{c\}>}$ to $\mathcal{I}$ (by Proposition 2, every such $N$ is also insufficient).

### 3.3 Finding a Seed

We now describe the procedure `findSeed`. The input is the latest identified MSR $M$, the set $\mathcal{M}$ of known MSRs, and the set $\mathcal{I}$ of known insufficient reductions. The output is a seed, i.e., a sufficient reduction $N$ such that $|N| < |M|$, or `null` if there is no seed. Let us denote by `CAND` the set of all *candidates* on a seed, i.e., $\texttt{CAND} = \{N \in \mathcal{R}_{\mathcal{A}} \,|\, |N| < |M|\}$. A brute-force approach would be to check individual reductions in `CAND` for sufficiency until a sufficient one is found, however, this can be practically intractable since $|\texttt{CAND}| = \sum_{i=1}^{|M|} \binom{|\Psi(\Delta) \cup \Psi(Inv)|}{i-1}$.

We provide three observations to prune the set `CAND` of candidates that need to be tested for being a seed. The first observation exploits the set $\mathcal{I}$ of already known insufficient reductions: no $N \in \mathcal{I}$ can be a seed. The second observation exploits the set $\mathcal{M}$ of already known MSRs. By the definition of an MSR, for every $M' \in \mathcal{M}$ and every $N$ such that $N \sqsubsetneq M'$, the reduction $N$ is necessarily insufficient and hence cannot be a seed. The third observation is stated below:

**Observation 1.** *For every sufficient reduction* $N \in \texttt{CAND}$ *there exists a sufficient reduction* $N' \in \texttt{CAND}$ *such that* $N \sqsubseteq N'$ *and* $|N'| = |M| - 1$.

*Proof.* If $|N| = |M| - 1$, then $N = N'$. For the other case, when $|N| < |M| - 1$, let $N = \mathcal{A}_{<D^N,I^N>}$ and $M = \mathcal{A}_{<D^M,I^M>}$. We construct $N' = \mathcal{A}_{<D^{N'},I^{N'}>}$ by adding arbitrary $(|M| - |N|) - 1$ simple clock constraint from $(D^M \cup I^M)\backslash(D^N \cup I^N)$ to $(D^N \cup I^N)$, i.e., $D^N \cup I^N \subseteq D^{N'} \cup I^{N'} \subseteq (D^M \cup I^M \cup D^N \cup I^N)$ and $|D^{N'} \cup I^{N'}| = |M| - 1$. By definition of `CAND`, $N' \in \texttt{CAND}$. Moreover, since $N \sqsubsetneq N'$ and $N$ is sufficient, then $N'$ is also sufficient (Proposition 2).

Based on the above observations, we build a set $\mathcal{C}$ of indispensable candidates on seeds that need to be tested for sufficiency:

$$\mathcal{C} = \{N \in \mathcal{R}_{\mathcal{A}} \,|\, N \notin \mathcal{I} \wedge \forall M' \in \mathcal{M}.\, N \not\sqsubseteq M' \wedge |N| = |M| - 1\} \tag{5}$$

The procedure `findSeed`, shown in Algorithm 3, in each iteration picks a reduction $N \in \mathcal{C}$ and checks it for sufficiency (via the verifier). If $N$ is sufficient, `findSeed` returns $N$ as the seed. Otherwise, when $N$ is insufficient, the algorithm first attempts to *enlarge* $N$ into an insufficient reduction $E$ such that $N \sqsubseteq E$. By Proposition 2, every reduction $N'$ such that $N' \sqsubseteq E$ is also insufficient, thus all these reductions are subsequently added to $\mathcal{I}$ and hence removed from $\mathcal{C}$ (note that this includes also $N$). If $\mathcal{C}$ becomes empty, then there is no seed.

The purpose of *enlarging* $N$ into $E$ is to quickly prune the candidate set $\mathcal{C}$. We could just add all the insufficient reductions $\{N' \mid N' \sqsubseteq N\}$ to $\mathcal{I}$, but note that $|\{N' \mid N' \sqsubseteq E\}|$ is exponentially larger than $|\{N' \mid N' \sqsubseteq N\}|$ w.r.t. $|E| - |N|$. The enlargement, shown in Algorithm 4, works almost dually to shrinking. Let $N = \mathcal{A}_{<D,I>}$. The algorithm attempts to one by one add the constraints from $\Psi(\Delta)\backslash D$ and $\Psi(Inv)\backslash I$ to $D$ and $I$, respectively, checking each emerged reduction for sufficiency, and keeping only the changes that preserve $\mathcal{A}_{<D,I>}$ to be insufficient.

### 3.4 Representation of $\mathcal{I}$ and $\mathcal{C}$

The final piece of the puzzle is how to efficiently manipulate with the sets $\mathcal{I}$ and $\mathcal{C}$. In particular, we are adding reductions to $\mathcal{I}$ and $\mathcal{C}$, removing reductions from $\mathcal{C}$, checking if a reduction belongs to $\mathcal{I}$, checking if $\mathcal{C}$ is empty, and picking a reduction from $\mathcal{C}$. The problem is that the size these sets can be expontential w.r.t. $|\Psi(\Delta) \cup \Psi(Inv)|$ (there are exponentially many reductions), and thus, it is practically intractable to maintain the sets explicitly. Instead, we use a symbolic representation. Given a TA $\mathcal{A}$ with simple clock constraints $\Psi(\Delta) = \{(e_1, \varphi_1), \dots, (e_p, \varphi_p)\}$ and $\Psi(Inv) = \{(l_1, \varphi_1), \dots, (l_q, \varphi_q)\}$, we introduce two sets $X = \{x_1, \dots, x_p\}$ and $Y = \{y_1, \dots, y_q\}$ of Boolean variables. Note that every valuation of the variables $X \cup Y$ one-to-one maps to the reduction $\mathcal{A}_{<D,I>}$ such that $(e_i, \varphi_i) \in D$ iff $x_i$ is assigned *True* and $(l_j, \varphi_j) \in I$ iff $y_j$ is assigned *True*.

The set $\mathcal{I}$ is gradually built during the whole computation of Algorithm 1. To represent $\mathcal{I}$, we build a Boolean formula $\mathbb{I}$ such that a reduction $N$ **does not** belong to $\mathcal{I}$ iff $N$ **does** correspond to a model of $\mathbb{I}$. Initially, $\mathcal{I} = \emptyset$, thus $\mathbb{I} = True$. To add an insufficient reduction $\mathcal{A}_{<D,I>}$ and all reductions $N$, $N \sqsubseteq \mathcal{A}_{<D,I>}$, to $\mathcal{I}$, we add to $\mathbb{I}$ the clause $(\bigvee_{(e_i,\varphi_i)\in\Psi(\Delta)\backslash D} x_i) \vee (\bigvee_{(l_j,\varphi_j)\in\Psi(Inv)\backslash I} y_j)$.

The set $\mathcal{C}$ is repeatedly built during each call of the procedure `findSeed` based on Eq. 5 and it is encoded via a Boolean formula $\mathbb{C}$ such that every model of $\mathbb{C}$ **does** correspond to a reduction $N \in \mathcal{C}$ :

$$\mathbb{C} = \mathbb{I} \wedge \bigwedge_{\mathcal{A}_{<D,I>}\in\mathcal{M}} ((\bigvee_{(e_i,\varphi_i)\in\Psi(\Delta)\backslash D} x_i) \vee (\bigvee_{(l_j,\varphi_j)\in\Psi(Inv)\backslash I} y_j)) \wedge \texttt{trues}(|\texttt{M}| - 1) \quad (6)$$

where $\texttt{trues}(|\texttt{M}| - 1)$ is a cardinality encoding forcing that exactly $|M| - 1$ variables from $X \cup Y$ are set to *True*. To check if $\mathcal{C} = \emptyset$ or to pick a reduction $N \in \mathcal{C}$, we ask a SAT solver for a model of $\mathbb{C}$. To remove an insufficient reduction from $\mathcal{C}$, we update the formula $\mathbb{I}$ (and thus also $\mathbb{C}$) as described above.

### 3.5 Related Work

Although the concept of minimal sufficient reductions (MSRs) is novel in the context of timed automata, similar concepts appear in other areas of computer

**Algorithm 3:** `findSeed`$(M, \mathcal{M}, \mathcal{I})$

**while** $\{N \in \mathcal{R}_{\mathcal{A}} \mid N \notin \mathcal{I} \wedge \forall M' \in \mathcal{M}.\, N \not\sqsubseteq M' \wedge |N| = |M| - 1\} \neq \emptyset$ **do**
    $N \leftarrow$ pick from $\{N \in \mathcal{R}_{\mathcal{A}} \mid N \notin \mathcal{I} \wedge \forall M' \in \mathcal{M}.\, N \not\sqsubseteq M' \wedge |N| = |M| - 1\}$
    **if** $N$ *is sufficient* **then** **return** $N, \mathcal{I}$
    **else** $\mathcal{I} \leftarrow \mathcal{I} \cup \{N' \in \mathcal{R}_{\mathcal{A}} \mid N' \sqsubseteq$ `enlarge`$(N)\ \}$
**return** `null`$, \mathcal{I}$

**Algorithm 4:** `enlarge`$(\mathcal{A}_{<D,I>})$

**foreach** $c \in (\Psi(\Delta) \cup \Psi(Inv)) \backslash (D \cup I)$ **do**
    **if** $c \in \Psi(\Delta)$ ***and*** $\mathcal{A}_{<D \cup \{c\}, I>}$ *is sufficient* **then** $D \leftarrow D \cup \{c\}$
    **if** $c \in \Psi(Inv)$ ***and*** $\mathcal{A}_{<D, I \cup \{c\}>}$ *is sufficient* **then** $I \leftarrow I \cup \{c\}$
**return** $\mathcal{A}_{<D,I>}$

science. For example, see minimal unsatisfiable subsets [15], minimal correction subsets [47], minimal inconsistent subsets [16,18], or minimal inductive validity cores [32]. All these concepts can be generalized as *minimal sets over monotone predicates (MSMPs)* [48,49]. The input is a reference set $R$ and a monotone predicate $\mathbf{P} : \mathcal{P}(R) \rightarrow \{1, 0\}$, and the goal is to find minimal subsets of $R$ that satisfy the predicate. In the case of MSRs, the reference set is the set of all simple constraints $\Psi(\Delta) \cup \Psi(Inv)$ and, for every $D \cup I \subseteq \Psi(\Delta) \cup \Psi(Inv)$, the predicate is defined as $\mathbf{P}(D \cup I) = 1$ iff $\mathcal{A}_{<D,I>}$ is sufficient. Many algorithms were proposed (e.g., [45,14,19,22,20,47,21,37,32,23]) for finding MSMPs for particular instances of the MSMP problem. However, the algorithms are dedicated to the particular instances and extensively exploit specific properties of the instances (such as we exploit reduction cores in case of MSRs). Consequently, the algorithms either cannot be used for finding MSRs, or they would be rather inefficient.

## 4 Synthesis of Relaxation Parameters

The main objective of this study is to make the target locations $L_T$ of a given TA $\mathcal{A} = (L, l_0, C, \Delta, Inv)$ reachable by only modifying the constants of simple constraints of $\mathcal{A}$. In the previous section, we presented an efficient algorithm to find a set of simple clock constraints $D \subseteq \Psi(\Delta)$ (1) (over transitions) and $I \subseteq \Psi(Inv)$ (2) (over locations) such that the target set is reachable when constraints $D$ and $I$ are removed from $\mathcal{A}$. In other words, $L_T$ is reachable on $\mathcal{A}_{<D,I>}$. Consequently, a verifier generates a finite run $\rho'_{L_T} = (l_0, \mathbf{0}) \rightarrow_{d_0} (l_1, v_1) \rightarrow_{d_1} \ldots \rightarrow_{d_{n-1}} (l_n, v_n)$ of $\mathcal{A}_{<D,I>}$ such that $l_n \in L_T$. Let $\pi'_{L_T} = l_0, e'_1, l_1, \ldots, e'_{n-1}, l_n$ be the corresponding path on $\mathcal{A}_{<D,I>}$, i.e., $\pi'_{L_T}$ is realizable on $\mathcal{A}_{<D,I>}$ due to the delay sequence $d_0, d_1, \ldots, d_{n-1}$ and the resulting run is $\rho'_{L_T}$. The corresponding path on the original TA $\mathcal{A}$ defined as in (4) is:

$$\pi'_{L_T} = M(\pi_{L_T}), \text{ and } \pi_{L_T} = l_0, e_1, l_1, \ldots, e_{n-1}, l_n, \tag{7}$$

While $\pi'_{L_T}$ is realizable on $\mathcal{A}_{<D,I>}$, $\pi_{L_T}$ is not realizable on $\mathcal{A}$ since $L_T$ is not reachable on $\mathcal{A}$. We present an MILP based method to find a relaxation valuation $\mathbf{r} : D \cup I \rightarrow \mathbb{N} \cup \{\infty\}$ such that the path induced by $\pi_{L_T}$ is realizable on $\mathcal{A}_{<D,I,\mathbf{r}>}$.

Given an automaton path $\pi = l_0, e_1, l_1, \ldots, e_{n-1}, l_n$ with $e_i = (l_{i-1}, \lambda_i, \phi_i, l_i)$ for each $i = 1, \ldots, n-1$, we introduce real valued delay variables $\delta_0, \ldots, \delta_{n-1}$ that represent the time spent in each location along the path. Since clocks measure the time passed since their last resets, for a fixed path, a clock on a given constraint (invariant or guard) can be mapped to a sum of delay variables:

$$\Gamma(x, \pi, i) = \delta_k + \delta_{k+1} + \ldots + \delta_{i-1} \text{ where } k = \max(\{m \mid x \in \lambda_m, m < i\} \cup \{0\}) \quad (8)$$

The value of clock $x$ equals to $\Gamma(x, \pi, i)$ on the i-th transition $e_i$ along $\pi$. In (8), $k$ is the index of the transition where $x$ is last reset before $e_i$ along $\pi$, and it is 0 if it is not reset. $\Gamma(0, \pi, i)$ is defined as 0 for notational convenience.

*Guards.* For transition $e_i$, each simple constraint $\varphi = x - y \sim c \in \mathcal{S}(\phi_i)$ on the guard $\phi_i$ is mapped to the new delay variables as:

$$\Gamma(x, \pi, i) - \Gamma(y, \pi, i) \sim c + p_{e_i,\varphi} \quad (9)$$

where $p_{e_i,\varphi}$ is a new integer valued relaxation variable if $(e_i, \varphi) \in D$, otherwise it is set to 0.

*Invariants.* Each clock constraint $\varphi = x - y \sim c \in \mathcal{S}(Inv(l_i))$ of the invariant of location $l_i$ is mapped to arriving (10) and leaving (11) constraints over the delay variables, since the invariant should be satisfied when arriving and leaving the location (and hence, due to the invariant convexity, also in the location).

$$\Gamma(x, \pi, i) \cdot \mathbf{I}(x \notin \lambda_i) - \Gamma(y, \pi, i) \cdot \mathbf{I}(y \notin \lambda_i) \sim c + p_{l_i,\varphi_i} \quad \text{if } i > 0 \text{(arriving)} \quad (10)$$

$$\Gamma(x, \pi, i+1) - \Gamma(y, \pi, i+1) \sim c + p_{l_i,\varphi_i} \quad \text{(leaving)} \quad (11)$$

where $\mathbf{I}$ is a binary function mapping *true* to 1 and *false* to 0, $p_{l_i,\varphi_i}$ is a new integer valued variable if $(l_i, \varphi_i) \in I$, otherwise it is set to 0.

Finally, we define an MILP (12) for the path $\pi$. The constraint relaxation variables $\{p_{l,\varphi} \mid (l, \varphi) \in I\}$ and $\{p_{e,\varphi} \mid (e, \varphi) \in D\}$ (integer valued), and the delay variables $\delta_0, \ldots, \delta_{n-1}$ (real valued) are the decision variables of the MILP.

$$\begin{aligned} \text{minimize} \quad & \sum_{(l,\varphi) \in I} p_{l,\varphi} + \sum_{(e,\varphi) \in D} p_{e,\varphi} \quad (12) \\ \text{subject to} \quad & (9) \text{ for each } i = 1, \ldots, n-1, \text{ and } x - y \sim c \in \mathcal{S}(\phi_i) \\ & (10) \text{ for each } i = 1, \ldots, n, \text{ and } x - y \sim c \in \mathcal{S}(Inv(l_i)) \\ & (11) \text{ for each } i = 0, \ldots, n-1, \text{ and } x - y \sim c \in \mathcal{S}(Inv(l_i)) \\ & p_{l,\varphi} \in \mathbb{Z}_+ \text{ for each } (l, \varphi) \in I, \text{ and } p_{e,\varphi} \in \mathbb{Z}_+ \text{ for each } (e, \varphi) \in D \end{aligned}$$

Let $\{p^\star_{l,\varphi} \mid (l, \varphi) \in I\}$, $\{p^\star_{e,\varphi} \mid (e, \varphi) \in D\}$, and $\delta^\star_0, \ldots, \delta^\star_{n-1}$ denote the solution of MILP (12). Define a relaxation valuation $\mathbf{r}$ with respect to the solution as

$$\mathbf{r}(l, \varphi) = p^\star_{l,\varphi} \text{ for each } (l, \varphi) \in I, \quad \mathbf{r}(e, \varphi) = p^\star_{e,\varphi} \text{ for each } (e, \varphi) \in D. \quad (13)$$

**Theorem 1.** *Let $\mathcal{A} = (L, l_0, C, \Delta, Inv)$ be a timed automaton, $\pi = l_0, e_1, l_1, \ldots, e_n, l_n$ be a finite path of $\mathcal{A}$, and $D \subset \Psi(\Delta)$, $I \subset \Psi(I)$ be guard and invariant constraint sets. If the MILP constructed from $\mathcal{A}$, $\pi$, $D$ and $I$ as defined in* (12) *is feasible, then $l_n$ is reachable on $\mathcal{A}_{<D,I,\mathbf{r}>}$ with $\mathbf{r}$ as defined in* (13).

*Proof sketch* Let $\{p^{\star}_{l,\varphi} \mid (l, \varphi) \in I\}$, $\{p^{\star}_{e,\varphi} \mid (e, \varphi) \in D\}$, and $\delta^{\star}_0, \ldots, \delta^{\star}_{n-1}$ be the optimal solution of MILP (12). Define clock value sequence $v_0, v_1, \ldots, v_n$ with respect to the path $\pi$ with $e_i = (l_{i-1}, \lambda_i, \phi_i, l_i)$ and the delay sequence $\delta^{\star}_0, \ldots, \delta^{\star}_{n-1}$ iteratively as $v_i = \mathbf{0}$ and $v_i = (v_{i-1} + \delta^{\star}_{i-1})[\lambda_i := 0]$ for each $i = 1, \ldots, n$. Along the path $\pi$, $v_i$ is consistent with $\Gamma(\cdot, \pi, i)$ (8) such that

$$a)\ v_i(x) = \Gamma(x, \pi, i).I(x \notin \lambda_i) \qquad \text{and} \quad b)\ v_i(x) + \delta^{\star}_i = \Gamma(x, \pi, i+1) \tag{14}$$

MILP (12) constraints and (14) imply that the path $M(\pi)$ that end in $l_n$ is realizable on $\mathcal{A}_{<D,I,\mathbf{r}>}$ via the delay sequence $\delta^{\star}_0, \ldots, \delta^{\star}_{n-1}$.

A linear programming (LP) based approach was used in [27] to generate the optimal delay sequence for a given path of a weighted timed automata. In our case, the optimization problem is in MILP form since we find an integer valued relaxation valuation ($\mathbf{r}$) in addition to the delay variables.

Recall that we construct relaxation sets $D$ and $I$ via Algorithm 1, and define $\pi_{L_T}$ (7) that reach $L_T$ such that the corresponding path $\pi'_{L_T}$ is realizable on $\mathcal{A}_{<D,I>}$. Then, we define MILP (12) with respect to $\pi_{L_T}$, $D$ and $I$, and define $\mathbf{r}$ (13) according to the optimal solution. Note that this MILP is always feasible since $\pi'_{L_T}$ is realizable on $\mathcal{A}_{<D,I>}$. Finally, by Theorem 1, we conclude that $L_T$ is reachable on $\mathcal{A}_{<D,I,\mathbf{r}>}$.

*Example 3.* For the TA shown in Fig. 1, Algorithm 1 generates $A_{<D,I>}$ with $D = \{(e_5, x \geqslant 25)\}$ and $I = \{(l_3, u \leqslant 26)\}$ such that $\pi = l_0, e_1, l_1, e_2, l_2, e_3, l_1, e_4, l_3, e_5, l_4$ is realizable on $A_{<D,I>}$. The MILP is constructed for $\pi$, $D$ and $I$ with decision variables $p_{e_5,x\geqslant 25}$, $p_{l_3,u\leqslant 26}$, $\delta_0, \delta_1, \delta_2, \delta_3, \delta_4$ and $\delta_5$ as in (12). The solution is $p_{e_5,x\geqslant 25} = 3$, $p_{l_3,u\leqslant 26} = 5$, and the delay sequence is $9, 4, 0, 9, 9, 0$. Consequently, $l_4$ is reachable on $A_{<D,I,\mathbf{r}>}$ with $\mathbf{r}(e_5, x \geqslant 25) = 3$ and $\mathbf{r}(l_3, u \leqslant 26) = 5$.

## 5 Case Study

We implemented the proposed reduction and relaxation methods in a tool called Tamus. We use UPPAAL for sufficiency checks and witness computation, and CBC solver from Or-tools library [50] for the MILP part. All experiments were run on a laptop with Intel i5 quad core processor at 2.5 GHz and 8 GB ram. The tool and used benchmarks are available at https://github.com/jar-ben/tamus.

As discussed in Section 1, an alternative approach to solve our problem (Problem 1) is to parameterize each simple clock constraint of the TA. Then, we can run a parameter synthesis tool on the parameterized TA to identify the set of all possible valuations of the parameters for which the TA satisfies the reachability property. Subsequently, we can choose the valuations that assign non-zero values (i.e., relax) to the minimum number of parameters, and out of these, we

**Table 1.** Results for the scheduler TA, where $|\Psi| = |\Psi(\Delta) \cup \Psi(I)|$ is the total number of constraints, $d = |D \cup U|$ is the minimum MSR size, $v$ is the number of reachability checks, $t$ is the computation time in seconds (including the reachability checks), and $c_m$ is the optimal cost of (12).

| Model | $\lvert\Psi\rvert$ | $d$ | $v$ | $t$ | $c_m$ | Model | $\lvert\Psi\rvert$ | $d$ | $v$ | $t$ | $c_m$ | Model | $\lvert\Psi\rvert$ | $d$ | $v$ | $t$ | $c_m$ |
|---|---|---|---|---|---|---|---|---|---|---|---|---|---|---|---|---|---|
| $\mathcal{A}_{(3,1,12)}$ | 11 | 2 | 33 | 0.18 | 6 | $\mathcal{A}_{(5,1,12)}$ | 16 | 3 | 120 | 0.63 | 10 | $\mathcal{A}_{(7,1,12)}$ | 19 | 3 | 120 | 0.63 | 11 |
| $\mathcal{A}_{(3,2,12)}$ | 17 | 1 | 13 | 0.13 | 13 | $\mathcal{A}_{(5,2,12)}$ | 24 | 1 | 42 | 0.35 | 13 | $\mathcal{A}_{(7,2,12)}$ | 28 | 1 | 95 | 0.72 | 13 |
| $\mathcal{A}_{(3,1,18)}$ | 16 | 3 | 61 | 0.37 | 9 | $\mathcal{A}_{(5,1,18)}$ | 23 | 4 | 149 | 0.90 | 16 | $\mathcal{A}_{(7,1,18)}$ | 28 | 5 | 313 | 1.87 | 25 |
| $\mathcal{A}_{(3,2,18)}$ | 24 | 1 | 40 | 0.40 | 6 | $\mathcal{A}_{(5,2,18)}$ | 35 | 1 | 57 | 0.58 | 6 | $\mathcal{A}_{(7,2,18)}$ | 42 | 1 | 70 | 0.74 | 6 |
| $\mathcal{A}_{(3,1,24)}$ | 21 | 4 | 97 | 0.65 | 12 | $\mathcal{A}_{(5,1,24)}$ | 31 | 6 | 327 | 2.16 | 24 | $\mathcal{A}_{(7,1,24)}$ | 38 | 7 | 709 | 4.76 | 35 |
| $\mathcal{A}_{(3,2,24)}$ | 32 | 1 | 80 | 0.85 | 16 | $\mathcal{A}_{(5,2,24)}$ | 47 | 2 | 169 | 1.80 | 31 | $\mathcal{A}_{(7,2,24)}$ | 57 | 2 | 201 | 2.21 | 21 |
| $\mathcal{A}_{(3,1,30)}$ | 26 | 5 | 141 | 1.05 | 15 | $\mathcal{A}_{(5,1,30)}$ | 39 | 7 | 541 | 4.17 | 31 | $\mathcal{A}_{(7,1,30)}$ | 48 | 10 | 1680 | 14.12 | 47 |
| $\mathcal{A}_{(3,2,30)}$ | 40 | 1 | 65 | 0.84 | 9 | $\mathcal{A}_{(5,2,30)}$ | 59 | 2 | 330 | 3.95 | 14 | $\mathcal{A}_{(7,2,30)}$ | 72 | 2 | 403 | 5.01 | 14 |

can choose the one with a minimum cumulative change of timing constants. In our experimental evaluation, we evaluate a state-of-the-art parameter synthesis tool called Imitator [9] to run such analysis. Although Imitator is not tailored for our problem, it allows us to measure the relative scalability of our approach compared to a well-established synthesis technique.

We used two collections of benchmarks: one is obtained from literature, and the other are crafted timed automata modeling a machine scheduling problem. All experiments were run using a time limit of 20 minutes per benchmark.

**Machine Scheduling** A scheduler automaton is composed of a set of paths from location $l_0$ to location $l_1$. Each path $\pi = l_0 e_k l_k e_{k+1} \ldots l_{k+M-1} e_{k+M} l_1$ represents a particular scheduling scenario where an intermediate location, e.g. $l_i$ for $i = k, \ldots, k+M-1$, belongs to a unique path (only one incoming and one outgoing transition). Thus, a TA that has $p$ paths with $M$ intermediate locations in each path has $M \cdot p + 2$ locations and $(M+1) \cdot p$ transitions. Each intermediate location represents a machine operation, and periodic simple clock constraints are introduced to mimic the limitations on the corresponding durations. For example, assume that the total time to use machines represented by locations $l_{k+i}$ and $l_{k+i+1}$ is upper (or lower) bounded by $c$ for $i = 0, 2, \ldots, M-2$. To capture such a constraint with a period of $t = 2$, a new clock $x$ is introduced and it is reset and checked on every $t^{th}$ transition along the path, i.e., for every $m \in \{i \cdot t + k \mid i \cdot t \leqslant M - 1\}$, let $e_m = (l_m, \lambda_m, \phi_m, l_{m+1})$, add $x$ to $\lambda_m$, set $\phi_m := \phi_m \wedge x \leqslant c$ ($x \geqslant c$ for lower bound). A periodic constraint is denoted by $(t, c, \sim)$, where $t$ is its period, $c$ is the timing constant, and $\sim \in \{<, \leqslant, >, \geqslant\}$. A set of such constraints are defined for each path to capture possible restrictions. In addition, a bound $T$ on the total execution time is captured with the constraint $x \leqslant T$ on transition $e_{k+M}$ over a clock $x$ that is not reset on any transition. A realizable path to $l_1$ represents a feasible scheduling scenario, thus the target set is $L_T = \{l_1\}$. We have generated 24 test cases. A test case $\mathcal{A}_{(c,p,M)}$ represents a timed automaton with $c \in \{3, 5, 7\}$ clocks, and $p \in \{1, 2\}$ paths with $M \in \{12, 18, 24, 30\}$ intermediate locations in each path. $R_{c,i}$ is the set of

**Table 2.** Experimental results for the benchmarks, where $|\Psi|$, $d$, $v$ $t$ and $c_m$ are as defined in Table 1, $|\Psi^u|$ is the number of constraints considered in the analysis and $m$ is the number of mutated constraints. $t^I$, $t^{IT}$, $t^{Ic}$ and $t^{ITc}$ are the Imitator computation times, where $c$ indicates that the early termination flag ("counterexample") is used, otherwise the largest set of parameters is searched, and $T$ indicates that only the constraints from the MSR identified by Tamus are parametrized, otherwise all constraints from $\Psi^u$ are parametrized. *to* shows that the timeout limit is reached (20 min.). We ran the Imitator with the flag "incl". Note that when run with the flag "merge", the performance of Imitator increases on 2 benchmarks, however, it decreases on other 2 benchmarks.

| Model | Source | Spec. | $\lvert\Psi\rvert$ | $\lvert\Psi^u\rvert$ | $d$ | $m$ | $v$ | $t$ | $c_m$ | $t^I$ | $t^{IT}$ | $t^{Ic}$ | $t^{ITc}$ |
|---|---|---|---|---|---|---|---|---|---|---|---|---|---|
| accel1000 | [11][35] | reach. | 7690 | 13 | 2 | 3 | 22 | 1.83 | - | 182.5 | 2.08 | 1.77 | 1.03 |
| CAS | [2] | reach. | 18 | 18 | 2 | 9 | 46 | 0.31 | 16 | 0.75 | 0.11 | 0.09 | 0.01 |
| coffee | [12] | reach. | 10 | 10 | 2 | 3 | 18 | 0.07 | 14 | 0.008 | 0.002 | 0.007 | 0.003 |
| Jobshop4 | [1] | reach. | 64 | 48 | 5 | 5 | 272 | 1.99 | - | to | 949.5 | to | 942.3 |
| Pipeline3-3 | [41] | reach. | 41 | 41 | 1 | 12 | 42 | 0.37 | - | to | 0.08 | to | 0.05 |
| RCP | [28] | reach. | 42 | 42 | 1 | 11 | 181 | 2.51 | - | to | 0.02 | 24.23 | 0.02 |
| SIMOP3 | [8] | reach. | 80 | 80 | 6 | 40 | 903 | 10.65 | - | to | 7.26 | to | 0.49 |
| Fischer | [36] | safety | 24 | 16 | 1 | 0 | 14 | 0.08 | - | to | to | 0.21 | 0.01 |
| JLR13-3tasks | [40][13] | safety | 42 | 36 | 1 | 0 | 40 | 0.41 | - | to | 2.60 | 0.05 | 0.08 |
| WFAS | [24][31] | safety | 32 | 24 | 1 | 0 | 10 | 0.08 | - | 16.20 | 0.01 | 0.03 | 0.006 |

periodic restrictions defined for the $i^{th}$ path of an automaton with $c$ clocks:

$$R_{3,1} = \{(2,11,\geqslant),(3,15,\leqslant)\} \qquad R_{3,2} = \{(4,17,\geqslant),(5,20,\leqslant)\}$$
$$R_{5,1} = R_{3,1} \cup \{(4,21,\geqslant),(5,25,\leqslant)\} \qquad R_{5,2} = R_{3,2} \cup \{(8,33,\geqslant),(9,36,\leqslant)\}$$
$$R_{7,1} = R_{5,1} \cup \{(6,31,\geqslant),(7,35,\leqslant)\} \qquad R_{7,2} = R_{5,2} \cup \{(12,49,\geqslant),(12,52,\leqslant)\}$$

Note that $\mathcal{A}_{(c,2,M)}$ emerges from $\mathcal{A}_{(c,1,M)}$ by adding a path with restrictions $R_{c,2}$.

Table 1 shows results achieved by Tamus on these models. Tamus solved all models and the hardest one $\mathcal{A}_{(7,1,30)}$ took only 14.12 seconds. As expected, the computation time $t$ increases with the number $|\Psi|$ of simple clock constraints in the model. Moreover, the computation time highly correlates with the size $d$ of the minimum MSR. Especially, if we compare two generic models $\mathcal{A}_{(c,1,M)}$ and $\mathcal{A}_{(c,2,M)}$, although $\mathcal{A}_{(c,2,M)}$ has one more path and more constraints, Tamus is faster on $\mathcal{A}_{(c,2,M)}$ since it quickly converges to the path with smaller MSRs.

Imitator solved $\mathcal{A}_{(3,1,12)}$, $\mathcal{A}_{(3,2,12)}$, $\mathcal{A}_{(3,1,18)}$, and $\mathcal{A}_{(5,1,12)}$ within 0.08, 0.5, 61, and 67 seconds, and timeouted for the other models. In addition, we run Imitator with a flag "counterexample" that terminates the computation when a satisfying valuation is found. The use of this flag reduced the computation time for the aforementioned cases, and it allowed to solve two more models: $\mathcal{A}_{(3,2,18)}$ and $\mathcal{A}_{(5,2,12)}$. However, using this flag, Imitator often did not provide a solution that minimizes the number of relaxed simple clock constraints.

**Benchmarks from Literature** We collected 10 example models from literature that include models with a safety specification that requires avoiding a set of locations $L_A$, and models with a reachability specification with a set of target locations $L_T$ as considered in this paper. In both cases, the original models satisfy the given specification. For the first case, we define $L_A$ as the target set and apply our method. Here, we find the minimal number of timing constants that should be changed to reach $L_A$, i.e., to violate the original safety specification. For the second case, inspired from mutation testing [2], we change a number of constraints on the original model so that $L_T$ becomes unreachable. Eight of the examples are networks of TAs, and while a network of TAs can be represented as a single product TA and hence our approach can handle it, Tamus currently supports only MSR computation for networks of TA, but not MILP relaxation.

The results are shown in Table 2. Tamus computed a minimum MSR for all the models and also provided the MILP relaxation for the non-network models. Note that the bottle-neck of our approach is the MSR computation and especially the verifier calls; the MILP part always took only few milliseconds (including models from Table 1), thus we believe that it would be also the case for the networks of TAs. The base variant of Imitator that computes the set of all satisfying parameter valuations solved only 4 of the 10 models. When run with the early termination flag, Imitator solved 3 more models, however, as discussed above, the provided solutions might not be optimal. We have also evaluated a combination of Tamus and Imitator. In particular, we first run Tamus to compute a minimum MSR $\mathcal{A}_{<D,I>}$, then parameterized the constraints $D \cup I$ in the original TA $\mathcal{A}$, and run Imitator on the parameterized TA. In this case, Imitator solved 9 out of 10 models. Moreover, we have the guarantee that we found the optimal solution: the MSR ensures that we relax the minimum number of simple clock constraints, and Imitator finds all satisfying parameterizations of the constraints hence also the one with minimum cumulative change of timing constants.

**Conclusion** In this work, we proposed the novel concept of a minimum MSR for a TA, that is a minimum set of simple constraints that need to be relaxed to satisfy a reachability specification. We developed efficient methods to find a minimum MSR, and presented an MILP based solution to tune these constraints. Our analysis on benchmarks showed that our tool Tamus can generate a minimum MSR within seconds even for large systems. In addition, we compared our results with Imitator and observed that Tamus scales much better. However, Tamus minimizes the cumulative change of the constraints from a minimum MSR by considering a single witness path. If the goal is to find a minimal relaxation globally, i.e., w.r.t. all witness paths for the MSR, we recommend to use the combined version of Tamus and Imitator, i.e., first run Tamus to find a minimum MSR, parametrize each constraint from the MSR and run Imitator to find all satisfying parameter valuations, including the global optimum.

**Acknowledgements** This research was supported in part by ERDF "Cyber-Security, CyberCrime and Critical Information Infrastructures Center of Excellence" (No. CZ.02.1.01/0.0/0.0/16_019/0000822) and in part by the European Union's Horizon 2020 research and innovation programme under the Marie Sklodowska-Curie grant agreement No. 798482.
